# Total Energy Expenditure and Body Composition in Two Free-Living Sympatric Lemurs

**DOI:** 10.1371/journal.pone.0009860

**Published:** 2010-03-25

**Authors:** Bruno Simmen, Françoise Bayart, Hanta Rasamimanana, Alexandre Zahariev, Stéphane Blanc, Patrick Pasquet

**Affiliations:** 1 UMR 7206, Eco-anthropologie et Ethnobiologie, Centre National de la Recherche Scientifique and Muséum National d'Histoire Naturelle, Brunoy, France; 2 Ecole Normale Supérieure, Université d'Antananarivo, Antananarivo, Madagascar; 3 UMR 7178, Institut Pluridisciplinaire Hubert Curien, Centre National de la Recherche Scientifique and Université Louis Pasteur, Strasbourg, France; University of Queensland, Australia

## Abstract

**Background:**

Evolutionary theories that account for the unusual socio-ecological traits and life history features of group-living prosimians, compared with other primates, predict behavioral and physiological mechanisms to conserve energy. Low energy output and possible fattening mechanisms are expected, as either an adaptive response to drastic seasonal fluctuations of food supplies in Madagascar, or persisting traits from previously nocturnal hypometabolic ancestors. Free ranging ring-tailed lemurs (*Lemur catta*) and brown lemurs (*Eulemur* sp.) of southern Madagascar have different socio-ecological characteristics which allow a test of these theories: Both gregarious primates have a phytophagous diet but different circadian activity rhythms, degree of arboreality, social systems, and slightly different body size.

**Methodology and Results:**

Daily total energy expenditure and body composition were measured in the field with the doubly labeled water procedure. High body fat content was observed at the end of the rainy season, which supports the notion that individuals need to attain a sufficient physical condition prior to the long dry season. However, ring-tailed lemurs exhibited lower water flux rates and energy expenditure than brown lemurs after controlling for body mass differences. The difference was interpreted to reflect higher efficiency for coping with seasonally low quality foods and water scarcity. Daily energy expenditure of both species was much less than the field metabolic rates predicted by various scaling relationships found across mammals.

**Discussion:**

We argue that low energy output in these species is mainly accounted for by low basal metabolic rate and reflects adaptation to harsh, unpredictable environments. The absence of observed sex differences in body weight, fat content, and daily energy expenditure converge with earlier investigations of physical activity levels in ring-tailed lemurs to suggest the absence of a relationship between energy constraints and the evolution of female dominance over males among lemurs. Nevertheless, additional seasonal data are required to provide a definitive conclusion.

## Introduction

Malagasy primates have physiological and behavioral features that correspond to energy conservation mechanisms, such as low basal metabolic rate (BMR), sun-bathing behavior, and in some nocturnal species, heterothermy, daily torpor and fattening [1]–[5]. From an evolutionary perspective, two distinct hypotheses have been offered to explain this energy pattern. From an adaptationist perspective, harsh ecological conditions that prevail in Madagascar, such as food shortage lasting for several consecutive months and erratic climate, would have driven lemur biology toward such energy saving mechanisms [1], [2], [4]. Lack of fruits for long periods, in particular, would explain why there are few obligate frugivores (i.e. with strategies of ‘high energy input/high energy crop’; [6]) among Malagasy lemur communities in contrast to the forest primate assemblages in different continents [7]–[9]). Seasonal energy constraints on lemurs would also have selected for highly seasonal reproductive cycles, with weaning occurring during the period of highest probability of fruit production (i.e. the rainy season), whatever the species body size within communities [4]. Finally, unpredictability of food supplies would have driven lemur social evolution toward strict female dominance over males with female priority in a feeding context in gregarious species, which is unusual among primates.

Other authors emphasize the major effect of phylogenetic inertia on the sociobiological characteristics shared by different lemur species. According to van Schaik and Kappeler [10], energy-conserving mechanisms and patterns of social dominance in lemurs are persisting traits derived from the nocturnal lifestyle of small-bodied, monogamous ancestors that had low BMR as found in other nocturnal or primitive mammal lineages, including marsupials. Such evolution would have occurred recently following the Holocene extinction of large diurnal raptors in Madagascar (among other megafauna), implying a transition from a nocturnal habit to a more diurnal lifestyle [10].

From the underlying assumptions of both hypotheses, low energy expenditure compared to haplorrhine primates or mammals with similar body size is expected in a broad range of Malagasy primates. Seasonal fattening might also be widespread among Malagasy prosimians, though not as marked as that found in the small-sized, heterothermous Cheirogaleids. Low basal energy needs are documented in a few species across different prosimian genera [3], [11]–[13]. However, there are few reference data on total energy expenditure (TEE) in free-living prosimians (and more generally in non-human primates), and there has been no investigation of body fat in prosimian species other than Cheirogaleid species. Furthermore, the TEE of any given species cannot be simply inferred from their BMR values. For instance, among folivorous species with limited daily behavioral activity and low quality diets, howler monkeys, guereza colobines and a few other species that derive most of their energy from fermentation of cell-wall polysaccharides do not have a particularly low relative BMR [14]–[16]. Referring to the scaling of field metabolic rate (FMR) to body mass determined with the doubly labeled water (DLW) method in mammals [17], free-living lemurs show either a low TEE (66% in *Lepilemur ruficaudatus*; [18]), or a TEE slightly above the regression line (120% in *Microcebus murinus*; [19]). Another study [20], [21] assessed energy spent by wild ring-tailed lemurs (*Lemur catta*) using the factorial method, a method in which the time allocated daily by an individual to different types of activity is converted into energy expenditure [22]. The physical activity level (PAL) of ring-tailed lemurs, i.e. the sum of all activity energy expenditures divided by resting metabolic rate was low and similar between males and females throughout most of the year [20], [21]. Given this limited set of data, the question of whether low energy expenditure is characteristic of Malagasy prosimians is still open.

The allometric exponent (0.734) obtained by Nagy [17] in the scaling relationship of FMR in mammals is close to the classic exponents determined for basal or resting metabolic rate in mammals (e.g. 0.75; [23]). Nagy concluded that a large part of the variation in FMR was due to variation in body mass, once the differences between endotherms and ectotherms was controlled, independent of the linear or non linear statistical models used to describe the data set. However, besides a major effect of body size on TEE, as revealed by earlier studies [24], [25], deviations from regression lines are large and could emphasize species' energy adaptations to ecological niches, types of diets and foraging strategies.

In the present paper, we report the total energy expenditure and body composition in 2 lemur species, the ring-tailed lemur (*Lemur catta*) and the brown lemur (*Eulemur* sp.), using the DLW method. This study stems partly from an earlier study of physical activity level (PAL) determined with the factorial method in *L. catta* troops followed for more than a decade [21], including groups that we investigated here with the isotopic method. Both species share a number of biological traits, like the lack of sexual dimorphism of body size, seasonal reproduction, group living as relatively large troops and a frugivorous-folivorous diet, although with distinct degrees of dietary diversity [26]–[34]. They differ in body size, diurnal versus cathemeral activity (24-h activity cycle in brown lemurs) and sociality: ring-tailed lemurs show strong hierarchical social relationships within and between sexes with female dominance over males, whereas there is little compelling evidence of dominance of one sex over the other in brown lemurs [1], [35]. Despite these social and ecological differences, the theories predict in both species low total energy expenditure (accounting for body size) as well as mechanisms to store energy before the long dry season during which lemurs cope with food scarcity and low-quality foods.

Choice of study period reflected a compromise between ethical considerations according to which gestating and lactating females should not be captured (from May to March, the overall dry season and part of the rainy season), and the need to carry out TEE measures during a potential critical period in terms of survival and reproductive success. It is hypothesized that these lemurs, like other Malagasy prosimians living in dry forest ecosystems, have to reach a sufficient physical condition prior to the dry season [3], [5], [31], [36]–[38]. Pereira et al. [36] argued that the rainy season is the major bottleneck for females that undergo full lactation. Females of reproductive age must store energy prior to the next breeding and gestation seasons that will extend over the driest months (see also [5]). Males may also be affected; they may have to store energy before the next short but intense competitive mating season. Given the close adjustment of the breeding cycles of both species to seasonal environmental changes, synchronized by photoperiod variation, the observations and experiments carried out in the present study took place at the end of the rainy season in March, before mating and gestation.

## Methods

### Ethics Statement

This research, involving non-human primates, has been approved by the scientific committee of the Muséum National d'Histoire Naturelle (BQR). In this study, all lemur manipulations have been conducted according to the international guidelines on health monitoring of non-human primate colonies by FELASA (Federation of European Laboratory Animal Sciences Associations). In accordance with the recommendations of Weatherall report, trapping, injections and blood sampling of the lemurs were conducted entirely under anesthesia using an hypnotic, so that the animals would not suffer nor recall the capture process. DLW experiments were approved by the Ministère de l'Environnement, Antananarivo, who delivered the license to collect and export biological samples (Autorisation de recherche n°11/MINENVEF/SG/DGEF/DPB/SCBLF/RECH, Direction Générale des Eaux et Forêts).

### Study site and species

The study took place in March 2006 in a gallery forest in a sub-arid area, South of Madagascar (Berenty Reserve, 25°0.29'S, 46°19.37'E). Ring-tailed lemurs are native to Berenty. The brown lemurs include a population of *Eulemur fulvus rufus* introduced in 1975 that later hybridized with a few introduced *Eulemur collaris*. Since then, the populations of ring-tailed lemurs and brown lemurs have grown to reach the high densities that may occur “naturally” in other dry forests. For the purpose of our study, this mixed *Eulemur* population is referred to as “brown lemurs”. In their distribution range, the two species live in sympatry in gallery forests dominated by tamarinds (*Tamarindus indica*) and a few other large tree species that are also the predominant plant species found at our study site. Details on the study site, forest composition, social relationships, diet and distribution of lemurs within this heterogeneous habitat can be found in several contributions published in Jolly et al. [39].

Ring-tailed lemur groups commonly range between 9 and 16 individuals, and brown lemur groups 8 and 13. Focal animals were selected from troops ranging in different areas of the forest. Focal lemur groups foraged in three main distinct forest types varying in structure and plant composition: Malaza closed canopy forest, edge of Malaza forest located at the tourist zone, and Ankoba secondary forest, bordered with ornamental plant species and crops. Adult females in each group gave birth during the prior austral spring. Troop history is documented in the case of *Lemur catta* [40]. Focal ring-tailed lemurs belonged to troops D1A, G3 and NA, the two former having been the focus of a long-term socio-ecological study [20]. Groups of ring-tailed lemurs were followed immediately before, during and after doubly labeled water experiments. Records were made of behavioral activities by scan sampling at 5-min intervals [41] adults that were involved in the doubly labeled water study. Behavioral observations were spread over the daytime phase in different continuous sessions between dawn and dusk. Individual behavioral data totaling 75h observations were pooled to assess the group activity budget. Brown lemurs have not received as much attention as ring-tailed lemurs, because of their cathemeral activity and relative lack of conspicuous external traits allowing quick individual recognition other than “male” or “female”. Quantitative data on brown lemur activity budgets during the study are not available. However, ad libitum observations were made on groups of brown lemurs that were selected to have home ranges overlapping with those of *L. catta*. Current knowledge of activity patterns ([29], [31], [32], Tarnaud and Simmen, unpublished results) will provide a basis for comparison.

Given a plant-based diet in both species, a broad picture of plant phenology (i.e. the feeding context) was obtained by assessing the proportions of trees and lianas (n = 157) bearing fruits, flowers, and leaves in Malaza forest.

### Doubly labeled water study

Total energy expenditure (TEE) was determined during a 3- to 4-day period by the two-point doubly labeled water (DLW) method [42].

Seventeen adult ring-tailed lemurs and 14 adult and one juvenile brown lemurs (balanced by sex) were captured using blowpipe darting with syringes filled with an anesthetic (ketamine 15 mg/kg). Only baseline and enrichment measures were available for some animals that could not be recaptured on time, yielding body composition results. Data for animals showing isotope inconsistencies due to contamination by ambient air of blood samples were discarded. As a result, in March, complete records for TEE and body composition of 10 ring-tailed and 11 brown lemurs (plus one juvenile) were obtained while body composition was assessed in 27 individuals.

After darting, animals were brought to a field experimental station. Physiological samples were collected and body mass measured. Blood samples from the inguinal vein were taken to assess baseline levels of natural isotope (^2^H and ^18^O) concentrations in the body fluids. A 0.2g/kg body mass premixed DLW dose was subcutaneously injected into the scapular region. The dose was composed of a mixture of 98% H_2_^18^O (Rotem Industries Ltd., Israel) and 99.9% ^2^H_2_O (Cambridge Isotope Laboratories, Andover, MA, USA) and calculated to ensure an *in vivo* enrichment of about 255 and 992 ‰ for 18-oxygen and deuterium, respectively [δ ‰ (delta per mil) = (R_sample_/R_standard_ – 1) * 1000 with R being the ratio ^2^H/^1^H in case of hydrogen)]. The DLW dose was weighed to the nearest 0.1 mg, controlling for DLW-syringe weight prior to and after injection. Isotopic equilibration in body water was determined through a second blood sample collected at 2-h post-dose. During a feasibility study carried out in December 2005, where we submitted three animals to the DLW dosing procedure (these animals were later tested in March 2006), a third point was measured at 3-h post-DLW injection. This pilot study, together with previous data that we recorded on body composition and TEE in various mammals, led us to consider 2 hours as the appropriate lapse of time required for isotopic equilibration following injection.

Immediately after blood collection, blood was flame-sealed in glass capillaries, totaling 500 µL, until analysis by isotope ratio mass spectrometry. The animal, equipped with either a radio-collar or a simple cat collar (for individual identification), was released on the same day and at the same place where it was captured. Lemurs, which forage as cohesive groups, permanently included at least one individual with a radio-collar on, which ensured locating the focal group. The lapse of time the subject-animal was out of his group for experimentation was less than 8 hours. Following recommendations of Sapolsky and Share [43], animals were released as soon as they were judged sufficiently alert after recovery from anesthesia. Subsequent monitoring of released animals showed that they were able to rapidly join their group apparently without social disruption. On the third or fourth day (depending on the ease of darting animals), the animals were recaptured and a final blood sample was collected approximately at the same time as the DLW injection on the first day of capture, to complete DLW calculations. All collars were removed at the end of the experiment.

Isotope ratio ^18^O and ^2^H enrichment was measured in body water after cryodistillation of the blood. 0.1 µL of the treated samples was injected in an elemental analyzer (Flash HT, ThermoFisher) connected to a continuous flow isotope ratio mass spectrometer (Delta V, ThermoFisher). Samples were reduced to H_2_ and CO at 1400°C on glassy carbon. H_2_ and CO were further separated at 104°C on a GC column before sequential analysis of deuterium and 18-oxygen isotopic abundances. The results were scaled using two laboratory standards. Analyses were performed in quadruplicate and repeated if the SD exceeded 2 ‰ for deuterium and 0.5 ‰ for 18-oxygen. CO_2_ production was calculated according to Speakman [44] and converted to TEE using Wier's equation assuming a food quotient of 0.909 and 0.891 for ring-tailed lemurs and brown lemurs, respectively, based on proportions of macronutrients in seasonal diets of each species (as derived from Simmen et al. ([31], in prep.).

Total body water (TBW) is calculated from the 18-oxygen dilution space after correction by the factor 1.007 for isotope exchanges [45]. Lean body mass was derived from TBW assuming a hydration coefficient of 73.2% [46]. Fat mass is calculated from the difference between body mass and lean body mass.

## Results

Results for body weight and body composition are presented in Table 1, and total energy expenditure (TEE) and water flux rate in Table 2. An ANOVA was used to test the effect of species and sex on total body water, % body water, fat mass and % body fat. An analysis of covariance was used to test these categorical variables on TEE and water flux, with body mass as a covariate.

**Table 1:** Body composition of ring-tailed lemurs and brown lemurs at Berenty.

Ring-tailed lemurs							
Identity (sex)	Body weight	Date of capture	Total body water		Free fat mass	Fat mass	
	g		g	%	g	g	%
March 2006:							
G3F1 (F)	2158	3/14/06	1315	60.9	1796	362	16.8
G3F3* (F)	2206	3/17/06	1388	62.9	1896	310	14.1
G3F4 (F)	2122	3/17/06	1258	59.3	1718	404	19
NAF8 (F)	2236	3/25/06	1204	53.8	1647	591	26.5
*mean ±sdev:*	*2181±50*		*1291±79*	*59.2±3.9*	*1764±108*	*417±123*	*19.1±5.3*
G3M1* (M)	2410	3/14/06	1448	60.1	1979	431	17.9
G3M2 (M)	2438	3/14/06	1398	57.4	1910	528	21.7
G3M4 (M)	2628	3/17/06	1536	58.4	2099	529	20.2
G3M5 (M)	2504	3/17/06	1453	58	1985	519	20.7
G3M8 (M)	2380	3/21/06	1434	60.2	1959	421	17.7
D1M6 (M)	1770	3/19/06	1114	62.4	1522	248	14
D1M7* (M)	2088	3/19/06	1289	61.7	1761	327	15.7
NAM9 (M)	2646	3/25/06	1590	60.1	2172	474	17.9
*mean ±sdev:*	*2358±294*		*1408±149*	*59.9±1.9*	*1923±203*	*435±102*	*18.2±2.6*
December 2005:							
NAF8 (F)	1840	12/10/05	1276	69.4	1744	96	5.2
NAM9 (M)	2328	12/9/05	1601	68.8	2187	141	6.1

Brown lemurs							
Identity (sex)	Body weight	Date of capture	Total body water		Free fat mass	Fat mass	
	*g*		*g*	*%*	*g*	*g*	*%*
March 2006:							
EFV1 (F)	1806	3/19/06	1115	61.7	1523	283	15.7
EFV3 (F)	1582	3/23/06	947	59.9	1294	288	18.2
EFV4 (F)	1762	3/24/06	1088	61.8	1487	275	15.6
EFV5 (F)	1674	3/24/06	1022	61.1	1396	278	16.6
EFV6 (F)	1726	3/24/06	1101	63.8	1503	223	12.9
EFV7 (F)	2166	3/26/06	1338	61.8	1829	337	15.6
EFV2 juv. (F)	1110	3/22/06	660	59.5	902	208	18.8
*mean ±sdev* a *:*	*1786±202*		*1102±132*	*61.7±1.3*	*1505±180*	*281±37*	*15.8±1.7*
EFL1 (M)	1800	3/19/06	1107	61.5	1512	288	16
EFL2 (M)	1688	3/22/06	1042	61.7	1423	265	15.7
EFL3 (M)	1582	3/22/06	1000	63.2	1367	215	13.6
EFL4 (M)	2144	3/23/06	1230	57.4	1681	463	21.6
EFL5 (M)	2172	3/24/06	1282	59	1751	421	19.4
EFL6 (M)	1966	3/24/06	1085	55.2	1482	484	24.6
EFL7 (M)	2038	3/24/06	1225	60.1	1674	364	17.9
EFL8 (M)	1806	3/24/06	1051	58.2	1435	371	20.5
*mean ±sdev:*	*1900±214*		*1128±104*	*59.6±2.6*	*1541±142*	*359±96*	*18.7±3.6*
December 2005:							
EFL7 (M)	1743	12/13/05	-	-	-	-	-

High-ranking individuals within each sex among ring-tailed lemur troops are indicated by an asterisk*.

a : excluding juvenile.

**Table 2:** Total energy expenditure (TEE) and water flux rates of ring-tailed lemurs and brown lemurs at Berenty.

Ring-tailed lemurs				
Identity (sex)	Date of	Date of	TEE	Water flux rate
	capture	recapture	*kJ.d^−1^*	*ml.d ^−1^*
March 2006:				
G3F3* (F)	3/17/06	3/20/06	859	427
G3F4 (F)	3/17/06	3/21/06	680	352
NAF8 (F)	3/25/06	3/29/06	589	302
*mean females ±sdev:*			*709±137*	*361±63*
G3M1* (M)	3/14/06	3/18/06	440	354
G3M2 (M)	3/14/06	3/18/06	635	365
G3M5 (M)	3/17/06	3/21/06	497	131
G3M8 (M)	3/21/06	3/25/06	736	438
D1M6 (M)	3/19/06	3/23/06	460	286
D1M7* (M)	3/19/06	3/23/06	592	324
NAM9 (M)	3/25/06	3/29/06	771	322
*mean males ±sdev:*			*590±132*	*317±95*
December 2005:				
NAF8 (F)	12/10/05	12/13/05	475	307

Brown lemurs				
Identity (sex)	Date of	Date of	TEE	Water flux rate
	capture	recapture	*kJ.d^−1^*	*ml.d ^−1^*
March 2006:				
EFV1 (F)	3/19/06	3/22/06	477	422
EFV3 (F)	3/23/06	3/27/06	606	535
EFV4 (F)	3/24/06	3/27/06	695	602
EFV5 (F)	3/24/06	3/27/06	553	557
EFV7 (F)	3/26/06	3/29/06	791	638
EFV2 juvenile (F)	3/22/06	3/26/06	434	392
*mean females ±sdev* a *:*			*624±122*	*551±82*
EFL2 (M)	3/22/06	3/26/06	611	495
EFL3 (M)	3/22/06	3/26/06	582	402
EFL5 (M)	3/24/06	3/28/06	700	461
EFL6 (M)	3/24/06	3/27/06	622	398
EFL7 (M)	3/24/06	3/29/06	528	479
EFL8 (M)	3/24/06	3/28/06	540	487
*mean males ±sdev:*			*597±62*	*454±43*

High-ranking individuals within each sex among ring-tailed lemur troops are indicated by an asterisk*.

a : excluding juvenile.

### Body composition

There was a significant species effect on body weight, total body water and fat mass but no sex effect (Table 3). No species or sex effect on body fat proportion and percentage of body water was found. Although data on seasonal changes in body composition are limited to one male (NAM9) and one female (NAF8) ring-tailed lemur, fat mass proportion increased substantially across the rainy season (between December and March) without any significant change in lean mass (Table 1). In the single individual *E. fulvus* captured in both seasons (EFL7), body mass increased by 17%, *i.e.* similar to the increase in ring-tailed lemurs, with body fat proportion reaching 17.9% at the end of the rainy season (March; Table 1). This suggests that this individual underwent a fattening process similar to that in *Lemur catta*.

**Table 3:** Species and sex differences of body composition, daily energy expenditure (DEE), and water flux rate in ring-tailed lemurs and brown lémurs.

	Effect	F values	*df*	*p*
**Body weight and body composition**				
Body weight a, g				
	*Species*	F = 21.099	22	**0.0001**
	*Sex*	F = 2.456	22	0.13
	*Species x sex*	F = 0.119	22	0.73
Total body water a, g				
	*Species*	F = 21.665	22	**0.0001**
	*Sex*	F = 3.001	22	0.17
	*Species x sex*	F = 0.808	22	0.37
Fat mass a, g				
	*Species*	F = 7.809	22	**0.01**
	*Sex*	F = 1.608	22	0.21
	*Species x sex*	F = 0.622	22	0.43
% body water a				
	*Species*	F = 1.140	22	0.29
	*Sex*	F = 0.578	22	0.45
	*Species x sex*	F = 1.946	22	0.17
% fat mass a				
	*Species*	F = 1.149	22	0.29
	*Sex*	F = 0.577	22	0.45
	*Species x sex*	F = 1.948	22	0.17
**Energy output/water flux rate**				
DEE, kJ.d^−1^*				
	*Species*	F = 0.813	17	0.38
	*Sex*	F = 3.882	17	0.065
	*Species x sex*	F = 1.416	17	0.25
	*Body weight*	F = 7.133	17	**0.01**
Water flux, ml.d^−1^*				
	*Species*	F = 16.331	17	**0.001**
	*Sex*	F = 3 .729	17	0.07
	*Species x sex*	F = 0.212	17	0.65
	*Body weight*	F = 1.844	17	0.19

Tests run as Ancova with body weight as a covariate (*) or as Anova. Significant differences (with *p*<0.05) are in bold.

a *:* excluding juvenile (EFV2).

### Total energy expenditure and water flux

There was no significant independent species or sex effect on daily energy expenditure, using body weight as a covariate, although females tended to spend more energy than males (p = 0.065; Table 3). Body weight is an effective predictor of TEE. There was a significant independent species effect on water flux, with a lower value in ring-tailed lemurs compared with brown lemurs. No statistically significant sex effect on water flux was found (p = 0.07; Table 3).

**Figure 1:**
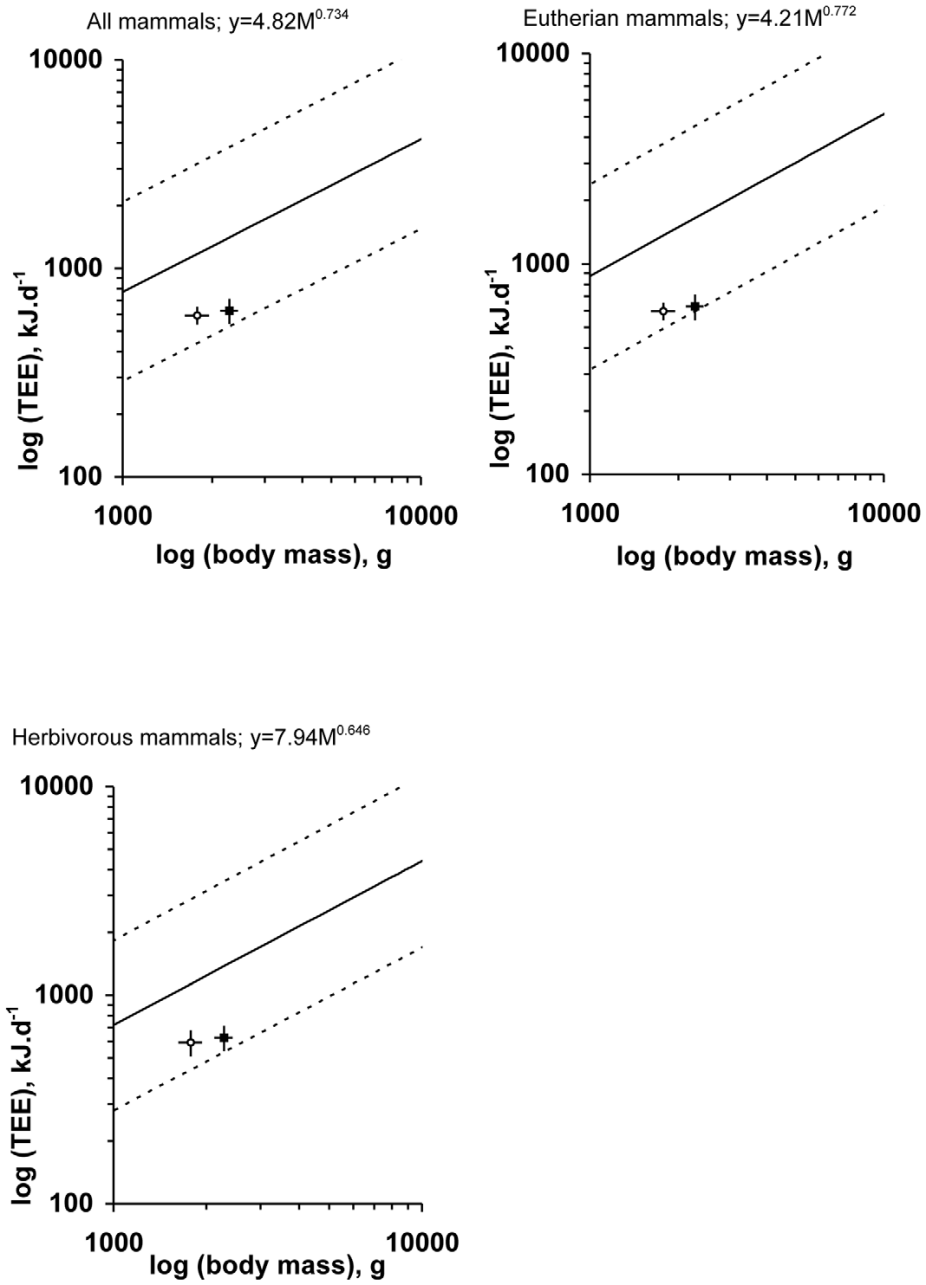
Total daily energy expenditure (TEE) of ring-tailed lemurs (black symbol) and brown lemurs (empty symbol) superimposed on the scaling of TEE across distinct phylogenetic and ecological grades of mammals. Equations, including 95% confidence interval of predicted TEE, are taken from [47]. TEE and body weight are means ±1.96sem.

Previous studies of field metabolic rate (FMR) determined by the DLW method provide a set of allometric exponents and coefficients [47] to account for differences in dietary grades, environments, and taxonomic classes among vertebrates; these can be used to make predictions on lemur FMR. We selected equations derived from mammal FMRs overall: eqn 1, FMR = 4.82M^0.734^, where M is body mass in g, 79 spp.; and those focused on eutherians: eqn 2, FMR = 4.21M^0.772^, 58 spp.; and herbivorous mammals: eqn 3, FMR = 7.94M^0.646^, 26 spp. On average, TEE is only 38–53% of the predicted mean FMR for ring-tailed lemurs and 44–60% for brown lemurs (Fig. 1).

The TEE/BMR ratio is a useful parameter to evaluate the surplus of energy devoted to costs other than maintenance of basal functions [22]. Rigorous records of oxygen consumption in *Eulemur fulvus* [11] show that minimum consumption occurs above 30°C, which corresponds to a BMR at 28% of the expected rate using Kleiber's equation. Since the author could not rule out the possibility that this extremely low level reflected a depressed rate due to high ambient temperature (up to 40°C), she suggested that basal rate may actually occur at lower temperatures (19–26°C), which yields a value that is 65% of the predicted BMR [11]. Unpublished results in two *Lemur catta* (McNab, pers. comm.) indicate very low BMR values that are 26 and 37% of prediction for basal metabolism using Kleiber's equation for comparison. However, it is unclear whether a depressed rate similar to that discussed for *Eulemur fulvus* might also have led to underestimating the true baseline energy demand of ring-tailed lemurs. In the absence of new data, TEE/BMR ratios are calculated using both 65% and 28% of predicted BMR values [23] in each species. As a result, average TEE/BMR ratios are between 1.8 and 4.1 in ring-tailed lemurs and between 2.0 and 4.7 in brown lemurs.

### Time budgets and feeding activity of ring-tailed lemurs

In ring-tailed lemurs, behavioral data pooled over the three focal troops yielded the following results: Traveling: 17.0%, Feeding 14.1%, Foraging 8.5%, Social activity (4.7%), Resting 53.7%, and Miscellaneous (2.0%). Ring-tailed lemurs showed low dietary diversity, with 9 out of 31 food items accounting for 75% of all feeding events recorded, a pattern consistent with previous reports at Berenty. Time spent feeding was mainly devoted to mature and young leaves (45.2%) and green and ripe fruits (35.4%), supplemented with flowers (9.4%) and miscellaneous items. The low proportion of fruit in the diet reflected plant phenology at this period, especially the limited number of forest trees and lianas bearing fruits (15%) or flowers (2%), and a few, introduced, tree species bearing ripe fruits at the forest edge. No comparable data are available for focal brown lemurs, but groups were frequently observed feeding on fruit species also eaten by *L. catta*.

## Discussion

**Figure 2:**
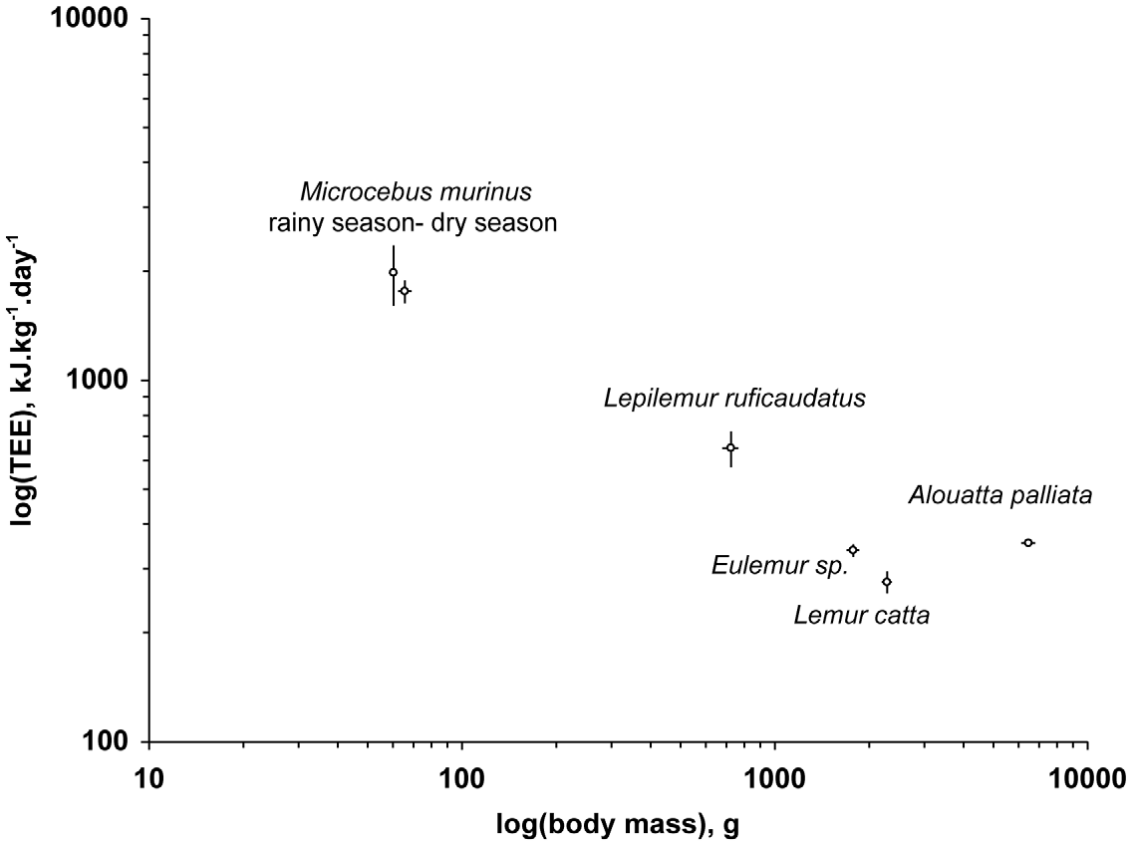
Daily energy expenditure per unit body weight in wild primates. Results (means ±sem) are derived from studies that used DLW in the field ([18], [19], [48], this study). *Lepilemur* n = 11; *Alouatta*, n = 6; *Microcebus* rainy season n = 7, dry season n = 23; *Eulemur* n = 12; *Lemur* n = 10).

Both adaptive and phylogenetic hypotheses to account for socio-biological characteristics of Malagasy lemurs, versus those of simians, predict the occurrence of energy conservation-mechanisms. The results of the present study, using the DLW method, clarify how ring-tailed and brown lemurs balance their energy needs. Referring to the scaling of energy expenditure to body mass in mammals studied with DLW [17], [47], the average TEE in both lemurs is within the range of variation of predicted field metabolic rate, but fall close to the lower limit of the 95% confidence interval. Compared with the few other nonhuman primate species studied with the DLW method including low-activity, folivorous species (Figure 2), the TEE values are lower after controlling for body mass differences [18], [19], [48]. Accordingly, one may consider ring-tailed lemurs and brown lemurs as “energy minimizers” at least during the late rainy season.

Could such economic lifestyles be explained by low metabolic rates? The extent to which the BMRs of ring-tailed lemurs and brown lemurs deviate from body size-related predictions remains a matter of debate since minimal consumption of oxygen was found to be between 65% of Kleiber's equation and as low as 26% ([11], [16], McNab, pers. comm.). Using both estimates of BMR, the ratio of TEE to basal metabolic rates for each species is around or above ratios of 2–3 that are commonly found in mammals [22], [25], [48], [49]. Low BMRs account for high TEE/BMR ratios found in other prosimian species tested with DLW. TEE values reach more than 3 times the BMR, while values <2 are noted in non-prosimian primates (howler monkeys and humans), and in marsupials [13], [17]–[19]. To disentangle relative effects of activity and maintenance costs on energy expenditure, one may also examine the activity budget. Summing the non-resting behaviors in *Lemur catta* during our study yields 46% of total scans recorded. Compared with other diurnal primates, especially those with similar dietary grades and BMR close to Kleiber (e.g. howler monkeys: annual 35%; [50]–[52]), this species does not appear inactive. The activity budget of brown lemurs, although not investigated here, is known from previous work, including comparative studies with ring-tailed lemurs and yet unpublished results ([29], [32], Tarnaud and Simmen, pers. comm.). The nocturnal activity of Berenty brown lemurs is limited to about 10% throughout the rainy season and increases during the dry season, whereas diurnal activity is maintained around 50%, which is similar to that found in ring-tailed lemurs ([40], [53]; Tarnaud and Simmen, unpublished results). These considerations are consistent with the idea that low FMR in ring-tailed lemurs and brown lemurs can be accounted for by low BMR.

From a functional standpoint, the low FMR in the larger *Lemur catta* compared to smaller brown lemurs could reflect differences of foraging strategies. Morphological adaptations of the digestive tract allow *Lemur catta* to meet part of its energy needs from the use of fibrous, ubiquitous leaves [54], as noted here. Brown lemurs, in contrast, digest fibers less efficiently [55], and free-ranging groups are able to forage over long distances to find ripe fruits when fruit is scarce [29], [31], [56]. In addition, brown lemurs are active both at canopy level and in the undergrowth [26], [29], whereas *Lemur catta* more frequently travels at ground level where they feed on herbaceous plants, thus reducing costs of vertical movements [32], [33].

### Water fluxes and water availability

The low water flux rate in *Lemur catta* compared with that of brown lemurs suggests mechanisms to conserve water. Limited water supply occurs under arid conditions like those faced by some ring-tailed lemur populations strictly living in Didiereaceae spiny forests and bush, where, in contrast, no *Eulemur* species occurs. Mechanisms to conserve water, such as oxidation of fat stored during the rainy season, which yields metabolic water, and low water evaporation, have been discussed in this way for sympatric lesser mouse lemurs (*Microcebus murinus*; [3], [19], [57]): Individuals undergoing periods of lethargy had lower water flux rates compared with normothermic individuals, although total energy expenditure did not differ between the two categories of individuals.

### Energy balance, reproductive condition and social dominance

Laboratory studies show that seasonal metabolic changes occur in ring-tailed lemurs and brown lemurs [36], [58], with variations in hair metabolism, levels of subcutaneous fat deposition, and body weight, among various physiological criteria. It is remarkable that in our sample the fat mass proportion of ring-tailed lemurs increased to an average of 18% (up to 26.5%) at the end of the rainy season, from low starting levels at about 6%. For comparison, wild baboons have only 1.9% fat mass [59] and captive baboons, marmoset or macaques average 8–10% [60]–[62]. We suspect there is a comparable fattening process in brown lemurs during the same period. Because the late wet season up to the end of the dry season is characterized by a drastic reduction of high quality food resources, especially ripe fruits in the dry forests of Madagascar ([28], [63], this study), fat storage will probably be an important source of energy for females and males alike. In addition, if one considers the rainy season as a major bottleneck for subsequent reproductive success, this hypothesis should not be restricted to females with putative high energy costs of gestation and early lactation. High fat levels in males, which have lean body mass similar to that of females, suggest that their energy expenditure during the months to come may be as high as that incurred by gestating females. During this period, males engage in costly activities, such as intense competition for access to fertile females and mate guarding [33], [64].

Should such energy conserving mechanisms be considered adaptive responses, or persisting traits associated with an ancestral nocturnal lifestyle (as proposed for several lemur characteristics; [10])? Low relative BMR and FMR are not necessarily associated with ancestral characteristics like small body size and nocturnality (e.g. *Microcebus murinus*; [3], [19]). Low FMR in ring-tailed lemurs and brown lemurs may reflect adaptive strategies in response to the particular constraints of the environment in which these species evolved. It is known that desert mammals have lower FMR than their non-desert relatives [17]. Such an environmental effect could make sense up to some point in *Lemur catta* because current populations are found not only in gallery forests but also in dry deciduous forests and Didiereaceae bush characterized by sub-arid climates with no access to fresh water, as well as in high mountain areas where temperatures below 0°C are frequent during winter [65]. The hypothesis that low energy expenditure is an adaptation to these particular conditions does not hold for brown lemurs, which do not currently inhabit such extreme ecosystems, presumably because of their more frugivorous habits and need for free water [29], [31], [55]. A plausible evolutionary explanation for similar low TEE in the two species emphasizes the unpredictability of edible food resources, which may have been a powerful, widespread selective pressure across Madagascar [4], [66], [67].

Concerning male versus female energy budgets, our data showing no sex difference in energy expenditure (or even a trend for females to spend more energy than males) converge with earlier results obtained on ring-tailed lemurs using the factorial method [20], [21], where PAL varied seasonally but appeared similar in females and males regardless of season. At first glance, these data seem inconsistent with the notion of food-related dominance relationships proposed by the Female need hypothesis [1] or by the Energy frugality hypothesis [4], both of which emphasize energy constraints for reproducing females in harsh environments. In addition, levels of stress that could modify energy expenditure in male *L. catta* vary over the year [68]. High levels of stress and sexual hormones in male *Eulemur rufus* occur not only during the mating period when sexual competition may be intense, but also during gestation [64]. Intra-sexual competition, male transfer between groups, challenging or maintaining rank position all generate energy costs that may equal those of females. However, our study was restricted by time and sample size, and PAL studies have some drawbacks. Thus, we cannot rule out the possibility that social dominance is associated with increased energy input or energy conservation at other seasons or that inter-individual variation in TEE within each sex was accidentally confounded with the particular social status of the focal animals. For example, Koyama et al. [69] found in a large sample at Berenty that, in autumn, body weight of ring-tailed lemur males varied across hierarchical ranks, suggesting different TEEs were mediated by social relationships. However, they did not find significant differences in body weight among females of different status.
